# Early Hyporegenerative Anemia Complicating Hemolytic Disease of the Newborn Secondary to Rhesus Alloimmunization

**DOI:** 10.7759/cureus.19603

**Published:** 2021-11-15

**Authors:** Catalina Acosta, Isra Idris, Rossana Romero, Lilian Ablan, Armyda Montoya Novoa, Awadelkarim Abdalaziz, Alexander Rodriguez

**Affiliations:** 1 Pediatrics, Woodhull Medical Center, New York, USA; 2 Pediatrics, Woodhull Medical Center, New york, USA; 3 Internal Medicine, Wayne State University, Detroit, USA; 4 Neonatalogy, Woodhull Medical Center, New York, USA

**Keywords:** neonatal indirect hyperbilirubinemia, rhesus alloimmunization, early hyporegenerative anemia, hemolytic disease, reticulocyte production and count

## Abstract

Rhesus hemolytic disease of the newborn is rarely found after the implementation of anti-D immunoglobulin prophylaxis. However, it may lead to cholestasis, elevated liver transaminases, hyperbilirubinemia, kernicterus, iron overload, and hyporegenerative anemia. Hyporegenerative anemia is characterized by low hemoglobin and reticulocyte count. It is typically recognized two to six weeks after birth. The etiology of this type of anemia is not identified yet, and treatment is controversial. We report a case of a neonate with rhesus hemolytic disease of the newborn with early hyporegenerative anemia that was noted on day seven of life. The available literature has described a similar age of onset, but after two weeks of life and not as early as on day seven of life as in our case. We treated this type of anemia with the standard of care management that includes phototherapy, intravenous immunoglobulin, and blood transfusions.

## Introduction

Rhesus hemolytic disease of the newborn rarely occurs after the implementation of anti-D immunoglobulin prophylaxis. It is three times more prevalent in Caucasians than in Blacks due to the different expressions of the Rhesus (*Rh*) gene [1]. It was first described in 1932 by Dr. Louis K. Diamond, who originated the term erythroblastosis fetalis after studying the blood smears of severely affected infants [2]. In 1940, Levine discovered the Rh blood group system, and the pathogenesis of this condition was finally confirmed by Chown et al. as being the result of the passage of Rh-positive red blood cells from the fetus to the mother’s circulation after transplacental hemorrhage [3].

A study developed by the United States and the United Kingdom in 1966 proved that administering anti-D immunoglobulin soon after delivery prevented the sensitization in D-negative women [3]. After the standardized use of Rh immunoglobulin in rhesus-negative women during the 1970s, the incidence of alloimmunization decreased dramatically from 14% to less than 0.2% at present [4,5].

In developing countries such as China, the prevention of alloimmunization with the use of Rh immunoglobulin and screening for antibodies is not well established. The distribution of the D-negative phenotype is around 3%-4%. Nevertheless, every D-negative pregnant woman has a 97% probability of carrying a D-positive fetus, increasing the chances of a severe hemolytic disease of the newborn requiring intrauterine transfusion or exchange transfusion after birth [4].

In the setting of prolonged hemolysis and repeated blood transfusions, cholestasis, iron overload, and late hyporegenerative anemia have been reported. Late hyporegenerative anemia is due to depressed erythropoiesis and is characterized by low reticulocyte count [6]. We report a case of a neonate with rhesus hemolytic disease with early hyporegenerative anemia that was noted at day seven of life, managed in the neonatal intensive care unit (NICU) at Woodhull Medical Center in Brooklyn, New York.

## Case presentation

We present a case of a newborn male who was delivered to a 35-year-old G3P2002 female at 37 weeks of gestation, admitted for induction of labor due to cholestasis. The pregnancy course was remarkable for GBS colonization, which was adequately treated, and Rh-negative status (A-), with anti-C and anti-D antibodies. Rhogam was administered in the previous two pregnancies, but not in the current one, due to high titers of anti-D antibodies, at 2048. 

The male infant was delivered with no complications. Apgar scores were 9 and 9 at one and five minutes of life, respectively. The newborn physical examination was within normal limits. On day one of life, screening laboratories confirmed Rh incompatibility (A-/O+; direct antiglobulin test (DAT) positive) and indirect hyperbilirubinemia with total bilirubin in high-risk zone surpassing photo level and reticulocytes of 8.89%. On physical examination, he was icteric with no hepatosplenomegaly. The patient was started on triple phototherapy, and immunoglobulin was administered at a dose of 1 g/kg. Subsequent laboratories showed increasing bilirubin and reticulocyte levels with downtrending hemoglobin and hematocrit, compatible with hemolytic disease of the newborn. A detailed representation of hematological indices and total bilirubin trend throughout the course of the illness is presented in Tables 1 and 2.

**Table 1:** Laboratory values: hemoglobin, hematocrit, and reticulocytes *After first transfusion; **after second transfusion

Day of life	Hb (g/dL) (normal range: 12.5–20.5 g/dL)	Hct (%) (normal range: 39%–63%)	Reticulocytes (%) (normal range: 1%–7%)
1	18.8	55	8.86
2	14.8	42.6	8.84
3	16.6	46.9	7.85
4	13.1	37.7	4.92
5	14.5	42	2.99
6	12.2	35.2	2.06
7	13	37.8	1.18
7	6.2	18	1.61
8	13*	36.4	-
8	15.9**	42.3	1.1
9	15.4	41.7	1
10	13.7	38.6	-
11	12.8	36.9	0.93
14	13	31.4	-
16	10	28.3	0.38

**Table 2:** Total bilirubin by days and hours of life

Day of life	Hours of life	Total bilirubin (mg/dL) (normal range: 4–12 mg/dL)
0	4	8.5
0	7	9.2
1	20	9.3
1	23	10.1
2	34	11.7
2	39	13.2
3	49	17.8
2	61	14.9
3	72	13.3
3	83	16
3	89	12.4
4	99	11.3
4	107	9.5
4	115	10.5
5	122	13.5
5	131	18.6
5	137	13.6
6	145	12.2
6	153	9.2
7	163	9.9
7	170	9.8
7	179	9.3
8	195	10.9
8	208	11.9
9	216	13.2
9	225	13.1
9	234	12.8
10	242	12.6
10	250	14.7
10	257	16.1
11	265	14.9
11	275	11.8
12	286	10.1
12	299	12.5
13	312	8.7
13	324	8.8
13	330	9.8
14	335	9.7
14	347	9.9
16	401	7.6

**Figure 1:**
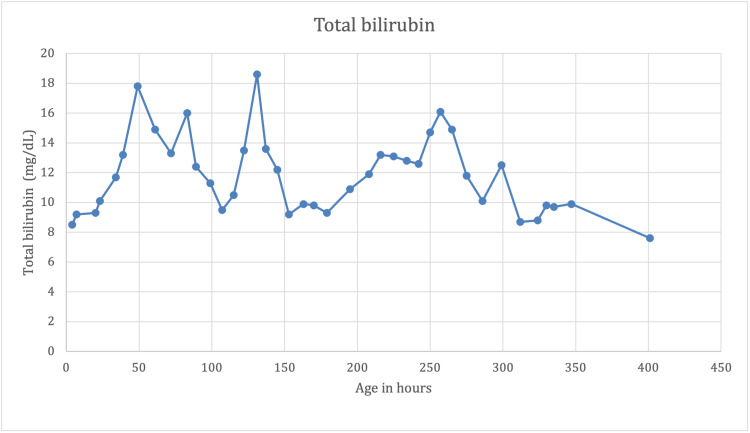
Total bilirubin (mg/dL) plotted against age in hours

**Figure 2:**
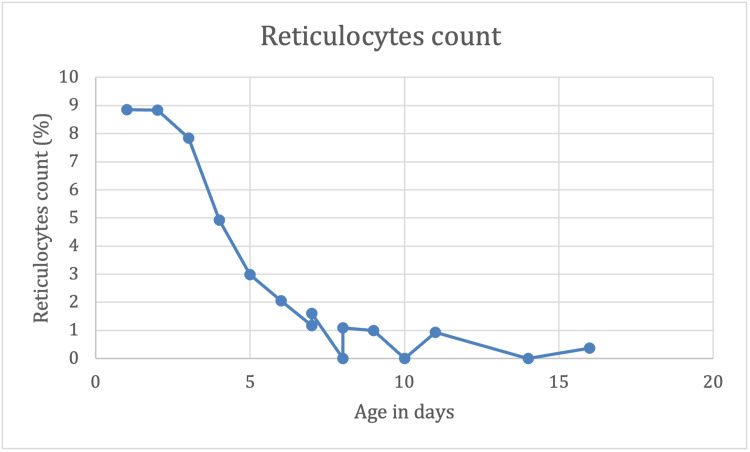
Reticulocyte count (%) plotted against age in days

On day three of life, despite continuous phototherapy, bilirubin level spiked from 13.2 to 17.8 mg/dL with a rate of rise of 0.46 mg/dL/hour. Quadruple phototherapy was added, and expectant management on partial exchange transfusion was considered. Over the course of the first eight days, the patient remained on continuous phototherapy with failed attempts to discontinue it due to persistently increasing rebound bilirubin levels and rate of rise of >0.2 mg/dL/hour. The graphs in Figures 1 and 2 demonstrate the trend of total bilirubin plotted against age in hours and reticulocyte count plotted against age in days, respectively. 

On day seven of life, the patient was tolerant to oral feeds at free demand with no tachypnea or tachycardia, and he presented with a further decrease in hemoglobin, reaching 6.2 g/dL; hematocrit of 18%; reticulocytes of 1.61%, which not concordant with the hemolytic process; and total bilirubin of 9.3 mg/dL. The patient received red blood cell (RBC) (O Rh-negative) transfusion at a dose of 20 mL/kg/day without complications. During the transfusion, intravenous fluids of dextrose 10% run at a target of 70 cc/kg/day and one dose of furosemide 1 mg/kg were provided. Posttransfusion hemoglobin was 13 g/dL, with a hematocrit of 36.4% and total bilirubin of 10.9 mg/dL. The patient received a second RBC (O Rh-negative) transfusion at a dose of 10 mL/kg over three hours. After the second transfusion, the patient had a hemoglobin of 15.9 g/dL and total bilirubin of 11.9 mg/dL. Subsequently, intravenous fluids were discontinued, and oral feeds with Similac 20 kcal were restarted with good tolerance.

On days 10 and 12 of life, attempts to discontinue phototherapy were unsuccessful due to the patient's total bilirubin, with a rate of rise of >0.24 mg/dL/hour and decreased hemoglobin and hematocrit. The patient continued double phototherapy until day 13 of life, when total bilirubin stabilized at 8.8 mg/dL, and it was determined to discontinue phototherapy completely. The rebound measurement of total bilirubin was 9.8 mg/dL, with a rate of rise of 0.14 mg/dL/hour. On day 14 of life, total bilirubin remained stable at 9.9 mg/dL, and it was decided to discharge the patient. On follow-up appointment on day 16 of life, total bilirubin was 7.6 mg/dL, hemoglobin 10.0 g/dL, hematocrit 28.3%, and reticulocytes 0.38%.

During the hospital stay, additional workup was done to rule out different etiologies of hyperbilirubinemia in the setting of persistent indirect bilirubin with a progressive decrease in reticulocytes (Figure 2). Peripheral blood smear, thyroid function tests, liver function tests, bile acids, G-6-PDH levels, and uridine 5'-diphospho (UDP)-glucuronosyltransferase 1A1 gene sequencing were found to be within the normal range.

## Discussion

The Rho(D) immunoglobulin or anti-D immunoglobulin is the gold standard for the prevention of rhesus (Rh) alloimmunization. It is obtained from pooled plasma selected from high titers of IgG antibodies to D-positive erythrocytes. Anti-D immunoglobulin contains polyclonal IgG speciﬁc to the Rh blood group system D polypeptide expressed on D-positive RBC. The mechanism by which Rh IgG prevents isoimmunization is not completely understood, but it is likely to suppress the immune response and antibody formation in Rh-incompatible individuals. Three main hypotheses remain predominant: the antigen clearance hypothesis, the FcγRIIB-mediated B-cell inhibition hypothesis, and the steric hindrance hypothesis [7,8].

The antigen clearance hypothesis states that IgG prevents the antibody response by accelerating the removal of antigens, such as RBC, from the circulation by the mononuclear phagocytic system, particularly from macrophages, before their recognition by the immune system. The FcγRIIB-mediated B-cell inhibition hypothesis proposes that the antigen-IgG complex co-ligates the B-cell receptor (BCR) with the inhibitory IgG receptor, FcγRIIB, on the B-cell surface and delivers a negative signal to inactivate antigen-specific B-cells. The steric hindrance hypothesis or epitope masking hypothesis suggests that IgG binds the antigen and blocks the specific epitope recognized by antigen-specific B-cells [7,9].

When alloimmunization is not prevented, the alloantibodies produced against the fetal erythrocytes remain in the neonatal circulation after birth for several months and can cause prolonged anemia. Anemia in hemolytic disease can present as early anemia (onset at birth to seven days of age) and late anemia, which is further divided into “late hyporegenerative anemia” and “late anemia of hemolytic disease.” Late hyporegenerative anemia has been described in the literature at week two to six of life, and it is characterized subsequently by low reticulocyte counts and is caused by depressed erythropoiesis [1,10].

Rh hemolytic disease of the newborn occurs when an Rh-negative pregnant woman is sensitized against Rh antigens present in the fetal blood. Maternal exposure to Rh D antigen can happen through vaginal delivery, fetal-maternal hemorrhage during pregnancy, abortion, ectopic pregnancy, placenta previa, invasive obstetric procedures, cesarean section, trauma, and transfusion with Rh-positive blood components [11]. The exposure can lead to the production of anti-D antibodies (IgG immunoglobulins) that cross the placenta and bind to the Rh D antigen present on fetal erythrocytes, leading to fetal hemolysis because of lysis by lysosomal enzymes from macrophages and natural killer lymphocytes, resulting in severe fetal anemia and hyperbilirubinemia, which can cause neurological damage or death [10,11].

In the United States, the recommendation for the administration of anti-D immunoglobulin was introduced in the 1970s. The current practice is to administer a single antenatal dose of 300 µg of anti-D immunoglobulin at 28 weeks of gestation, followed by a second dose within 72 hours after birth if newborn Rh D typing has identified the infant as Rh-positive [12]. The longer prophylaxis is delayed, the less it will be protective, but the patient may still receive some benefit from anti-D immunoglobulin as late as 28 days postpartum [13].

In cases of exposures larger than 30 mL of Rh D-positive fetal whole blood, which occurs in two to three of 1,000 deliveries, additional anti-D immunoglobulin is needed to prevent alloimmunization. The assessment of the volume of fetal-maternal hemorrhage to determine the amount of anti-D immunoglobulin required to prevent alloimmunization is paramount (rosette screen). A positive rosette test should be followed by a method that determines the percentage of fetal red blood cells in maternal circulation, such as the Kleihauer-Betke test or flow cytometry. In clinical situations in which fetal-maternal hemorrhage is >30 mL of fetal whole blood or 15 mL of fetal red cells, additional vials of Rh immunoglobulin can be administered at one time [12].

Compared to the previous description in literature, the presented case of Rh incompatibility with prolonged hospital course, in which the patient received intensive phototherapy and immunoglobulin, is associated with an apparent earlier hyporegenerative anemia. The case started presenting reduced reticulocyte count from the fourth day of life with reticulocytes of 4.92% (Figure 2). As reported in the literature, reticulocyte count would fall almost to 0% by this postnatal age of four to seven days [14]. However, in the setting of this patient with alloimmune hemolytic disease, the patient failed to start reticulocyte production when the hemoglobin deficit exceeded 2 g/dL compared with gestational age norms [15]. By day seven of life, this patient with alloimmune hemolytic disease required its first RBC transfusion as the hemoglobin fell to 6.2 mg/dL, with a hematocrit of 18% and reticulocytes of 1.61% (Figure 2). Hyporegenerative anemia is characterized by decreased erythropoiesis with diminished erythrocyte count that can be low or undetectable [6,9]. It has been reported that hyporegenerative anemia can present concomitantly with rhesus hemolytic disease, with limited literature available. Although the etiology of hyporegenerative anemia is still not clear, it has been linked with intramedullary destruction of normoblast due to Rh antibodies, erythropoietin deficiency, relative anemia due to the expanding intravascular volume of the growing neonate, and bone marrow suppression from intrauterine and postnasal transfusions. However, studies have reported that the erythropoietin deficiency component in hyporegenerative anemia in the setting of Rh incompatibility varies. The treatment protocols suggest the use of recombinant human erythropoietin (rHuEPO) for hyporegenerative anemia; however, studies have shown that it can be ineffective when anti-D titers are high [6,16].

One of the known complications of neonates with alloimmune hemolytic disease is iron overload. This is caused by the combination of prolonged hemolysis, treatment with multiple intrauterine blood transfusions (IUTs), and erythrocyte transfusions. Although iron is essential in early brain development, iron overload can cause damage to the heart, liver, and endocrine organs and increases susceptibility to infection by altering the immune response. The transfusions that are done usually raise the iron load and perpetuate the hyporegenerative state [6,17].

In severe cases of hemolytic disease of the newborn due to increased erythropoiesis, the suppression of other cell lines can occur, potentially causing leukopenia and thrombocytopenia. However, in the case of platelets and the rest of the cell lines, they remained within the normal parameters throughout the hospital stay [17].

On the other hand, late anemia of hemolytic disease is characterized by the active bone marrow to compensate for the shortened erythrocyte survival, which is reflected by elevated reticulocyte counts. This form of late anemia is thought to be due to a combination of continuing hemolysis by remaining antibodies, natural decline of the hemoglobin level, shortened survival of transfused erythrocytes, and the expanding intravascular volume of the growing neonate. Late anemia usually resolves by the third month of life; until then, erythrocyte transfusions may be necessary to treat postpartum anemia. Variations in the percentage of neonates requiring top-up transfusions may be explained by multiple factors: differences in transfusion guidelines and thresholds for erythrocyte transfusions [2,17]. In this case, the patient was not followed up, and as a result, the outcome of the possible late anemia could not be obtained.

Another complication of alloimmune hemolytic disease and elevated unconjugated bilirubin (free bilirubin) levels is neuronal injury. The degree of damage is linked to the concentration of free bilirubin and hydrogen ion (pH) in blood [18]. Permeation of the blood-brain barrier occurs when the capacity of blood to bind bilirubin is exceeded due to high free bilirubin concentration or when bilirubin binding sites on albumin are displaced by other substances such as sulfonamides. It is suggested that the mechanism of the blood-brain barrier to reduce the concentration of unconjugated bilirubin by ATP-dependent export by transporter molecule is disrupted by high levels of free bilirubin [19]. Neuronal damage has been linked to apoptosis and necrosis due to the disturbance of mitochondrial function and calcium intracellular homeostasis. The neuropathology of bilirubin-induced brain injury is complex as it includes a wide range of clinical presentations, including the classical kernicterus with athetoid cerebral palsy, impaired upward gaze, and deafness, as well as auditory neuropathy/dys-synchrony and subtle bilirubin-induced neurological dysfunction (BIND) [18]. During the hospital stay, the patient presented with no signs or symptoms of neurological alteration, and the neurological examinations were within the normal limits.

## Conclusions

Rhesus hemolytic disease of the newborn continues to occur despite anti-D immunoglobulin prophylaxis. The clinical severity of rhesus hemolytic disease of the newborn varies from mild to severe and depends on the amount of antibody that crosses the placenta. The challenge in the management of hyperbilirubinemia and hyporegenerative anemia requires a complex interplay of adequate screening of high-risk pregnancies, anticipating hyperbilirubinemia of neonates, and combining antenatal and postnatal management. In our case, hyporegenerative anemia was noted on day seven of life and was challenging as reticulocyte production did not increase despite the reduction in hemoglobin due to hemolysis. We treated this type of anemia with the standard of care management that includes phototherapy, intravenous immunoglobulin, and blood transfusions. However, more extensive studies are warranted to evaluate the causes of hyporegenerative anemia. The case also provided an opportunity to review the topic of hemolytic disease in the newborn secondary to Rh alloimmunization, but despite its low incidence, if undertreated, it can lead to neuronal injury with different clinical presentations.
